# Nurse managers and workplace violence: the mental health and well-being of nurses and personal care assistants in aged care

**DOI:** 10.1108/JHOM-10-2025-0722

**Published:** 2026-07-03

**Authors:** Jillian Cavanagh, Timothy Bartram, Patricia Pariona-Cabrera

**Affiliations:** School of Management, RMIT University, Melbourne, Australia; RMIT University, Melbourne, Australia

**Keywords:** Aged care, Violence, Nurses, Managers, Personal care assistants, Mental health, Well-being, Symbolic violence

## Abstract

**Purpose:**

The purpose of the study is to examine the impact of workplace violence on the mental health of nurse managers, nurses and personal care assistants.

**Design/methodology/approach:**

The study is qualitative and uses Conservation of Resources Theory and Theory of Practice as the combined theoretical framework to examine the narratives of 50 participants.

**Findings:**

There are serious mental health and well-being challenges of both nurse managers, nurses and personal care assistants, as sustained by multiple episodes of workplace violence. We found evidence of symbolic violence used by senior management and limited evidence of perceived formal and informal management supports to mitigate and manage workplace violence.

**Research limitations/implications:**

The study was limited to the state of Victoria in Australia. Non-ANMF members were not included as a comparison group in this study. Therefore, it was not possible to determine if the experiences raised by nurse managers, nurses and personal care assistants in aged care facilities were unique to this sector. While we interviewed managers, aged care workers from public, private and not-for-profit aged care facilities, we did not set out to compare the three sectors. We did not interview HR managers.

**Practical implications:**

HRM departments and managers in aged care facilities also need to ensure that health and safety compliance is followed in accordance with relevant legislation and employment agreements. It is the responsibility of nurse managers and nurses and personal care assistants to ensure that incidents of violence are reported, and measures taken to avoid further workplace violence incidents.

**Social implications:**

The care of an ageing population is important to society. When nurses and personal care assistants are supported in the workplace by nurse managers, they are more likely to provide better care to residents of aged care facilities.

**Originality/value:**

Our study contributes to the theoretical relevance of Hobfoll’s COR theory and Bourdieu’s Theory of Practice by demonstrating how the theories combined are relevant to contemporary workers and organisations within a specific context or sector.

## 1. Introduction

In recent times, there have been escalating concerns about nurses and personal care assistants (PCAs) in the aged care sector and how these workers endure poor working conditions, heavy workloads and workforce shortages (Bartram *et al*., 2023). In this study, the impact of workplace violence on the mental health and well-being of nurses and PCAs is an important challenge in aged care facilities because resident numbers are increasing and nurse and PCA numbers are declining (Pariona-Cabrera *et al*., 2020). This paper focuses on examining the impact of workplace violence on the mental health and well-being of nurses and PCAs and draws implications for nursing practices in aged carer facilities. In the final report of the Royal Commission into Aged Care Quality and Safety (2021) in Australia, there was a commitment to supporting “a highly skilled, well rewarded and valued aged care workforce … ” (p. 124). It is recognised that the aged care workforce “is vital to the success of any future aged care system”. However, “there are systemic workforce problems (in aged care facilities) that must be addressed” due to “poor employment conditions; lack of investment in workers; staff training; limited opportunities to progress or be promoted; and no career pathways” (p. 76).

The World Health Organization (2018) defines workplace violence as “any violent act, including physical or verbal violence directed toward people at work or on duty” (p. 1). Verbal violence can be related to verbal threats and expressions to cause harm, and physical violence may be pinching, hitting and biting. In the USA, 73% of all non-fatal workplace injuries are caused by incidents of violence across all industries in 2018 (U.S. Bureau of Labor Statistics, 2019). In Australia, more than 60% of healthcare workers experienced physical or verbal violence in 2021 (Safe Work Australia, 2023). According to Safe Work Australia, the health care and social assistance industries have the highest risk of workplace violence and aggression (Safe Work Australia, 2023).

Our study is significant due to the ageing population, the need for nurse managers, nurses and PCAs, and the levels of workplace violence that escalate mental health issues among the aged care workforce (Pariona-Cabrera *et al*., 2020). According to the Nursing and Midwifery Board of Australia (2025), there are 478,617 registered nurses (RN), enrolled nurses (EN) and combined EN and RN across Australia, representing the largest clinical workforce in the country. Despite the number of nurses increasing over the last decade, there is concern that the demand from an ageing population will eventually exceed supply, with a predicted shortfall of nurses being around 123,000 by 2030. In our study, we focus on the perspectives of nurse managers, nurses, and PCAs concerning the impact that workplace violence has on their mental health and well-being. We define mental health in the following way:

Mental health is a dynamic state of internal equilibrium which enables individuals to use their abilities in harmony with universal values of society. Basic cognitive and social skills; ability to recognize, express and modulate one’s own emotions, as well as empathize with others; flexibility and ability to cope with adverse life events and function in social roles; and harmonious relationship between body and mind represent important components of mental health which contribute, to varying degrees, to the state of internal equilibrium (Galderisi *et al*., 2015, p. 231).

There is a paucity of research in aged care facilities (Royal Commission into Aged Care Quality and Safety, 2021) despite the prevalence of incidents of violence, which is often considered part of nursing work (Pariona-Cabrera *et al*., 2020). More attention needs to be afforded to this cohort of workers who support vulnerable and an ever-ageing population (Shao *et al*., 2023). In our study, we examine how nurses and PCAs conserve and develop their resources and cope with symbolic violence from the hierarchy to help us plan and understand mechanisms that may better support nurses and PCAs. Our main research question is: What is the impact of workplace violence on nurse managers, nurses and PCAs in aged care facilities and how are such incidents managed?

We make two contributions to the nursing management literature. First, we provide new perspectives from nurse managers, nurses and PCAs about how the normalisation of violence in the aged care sector is shaped by senior management through symbolic violence, a decline in nurses and PCAs resources and their reluctance to challenge management constraints. Second, we contribute new knowledge from the perspectives of the participants about the systemic management failures (e.g. prioritising budget constraints, ignoring incidents of violence, discouraging incident reports, no psychological support and a culture of silence) that protect residents but are harmful to the mental health and well-being of nurses and PCAs.

## 2. Literature on aged care and incidents of violence

Aged care facilities provide care services for many older people who require assisted living services and provide accommodation and 24-h care for those who have greater care needs and choose or need to be cared for in an aged care facility (Aged Care Financing Authority, 2019). The Australian Government Department of Health (2021) has called for more organisational support for nurses and PCAs across this country to mitigate workplace violence. Workplace violence in aged care refers to any incident where workers in this sector contend that aged care in this country “produces a brutal corporeal labour regime that perpetuates the extreme exploitation of worker bodies” (p. 215). The study found, care for many aged clients is carried out by unqualified nurses and aged care organisations are responsible for fostering work environments of fear and aggression. Psychogeriatric nurses need to be trained and employed within aged care facilities if workplace violence is going to be reduced and the exploitation of workers, their mental health and well-being and improve the psychological safety of workers.

As a clinician researcher, Galik (2022) wrote about her own experiences related to long-term facilities in healthcare systems across the USA. She concluded that workers in aged care facilities are constantly dealing with violence from residents, their families, management and in some cases other staff. Galic calls for the hierarchy of aged care facilities to provide anti-violence interventions and ensure the safety of all workers who are employed in this sector. In another study in the USA, Lombardi *et al*. (2024) examined data from a Bureau of Labor Statistics Survey of Occupational Injuries and Illness between 2011 and 2022 across various healthcare facilities and revealed that over that time, there had been a 30% increase in workplace violence against healthcare workers. The authors argue that systemic issues effectively increase incidents of violence due to organisational complacency, which negatively impacts on workers. Many facilities experience daily staff shortages, work with poor organisational resources and ineffective management that fail to protect workers. The author contends that aged care facilities are highly vulnerable to verbal and physical violence and more needs to be done to understand the issues and develop interventions that better support workers. In Portugal, Barros *et al*. (2022) carried out a cross-sectional study comprising 276 healthcare workers, of which 59.1% were nurses employed in various healthcare settings and assessed predictors of vicarious violence and emotional issues that impacted healthcare workers. The authors identified high work demands, excessive work hours and challenging employment relations as psychological risks for workers. This study highlighted the need for organisations to be more proactive in reducing the risks associated with violence.

While the aforementioned studies make important contributions, they remain unresolved debates in the literature, as we have not found any articles that applaud the hierarchy within the aged care sector. Nurses’ mental health remains impacted by workplace violence, job stress and burnout (Bartram *et al*., 2023). Watson and Hatcher (2021) argue that aged care hierarchies continue to fail workers, such as nurse managers, nurses and PCAs, in favour of administrative responsibilities and the operations of facilities. The hierarchy within the aged care sector exercises symbolic violence to control workers, which can only contribute to growing tensions between hierarchical management, nurse managers, nurses and PCAs. In these demanding workplace environments, effective prevention of the negative consequences of workplace violence on workers is important, given the high rates of attrition and shortage of nurses, and more needs to be known about the conditions for these critical workers (Shao *et al*., 2023).

## 3. Theoretical framework

We employ two theoretical frameworks in this paper to generate deeper explanatory insights into what is going on in aged care facilities. Conservation of resources theory (COR) (Hobfoll, 1989) provides a lens to better understand the resources that nurse managers, nurses and PCAs gather to conserve and protect themselves to overcome difficult work situations, such as workplace violence and management constraints and the impact on their mental health. We considered other theoretical frameworks, but they did not provide a thorough mechanism to examine resource depletion to the same degree as COR theory. Even though COR theory has not been widely used to seek deeper insights, we justify and strengthen the framework by combining it with Bourdieu’s Theory of Practice (ToP) (1977). Both theories recognise deficient hierarchical support, which validates the application of COR and ToP. The nurse managers, nurses and PCAs of our study work under conditions where the staff/resident ratios are inadequate, there are constant failings in management support, and HRM is external to aged care facilities, leaving workers with few to no avenues for organisational help. COR theory emphasises organisational and context pressures and also explains how workers can be subjected to loss spirals, which leave them vulnerable to additional resource losses (Hobfoll, 2001). The theory also accounts for how workers enter loss spirals: when depleted staff have fewer resources to cope, they become even more susceptible to further loss, reduced well-being and diminished capacity to provide safe, person-centred care. Importantly, COR theory acknowledges the impact of organisational constraints and analysing how they impact staff health and well-being. Three COR theory mechanisms, including resource gain, resource caravans and gain spirals, are explained in the following paragraphs.

Resource gain refers to a mechanism in which individuals aim to obtain and retain resources to cope with adverse work events and any negative effects (Hobfoll, 1989). Resource gain may help nurse managers, nurses and PCAs manage the negative effects of workplace violence on their mental health by preserving resources that protect their self-esteem and enhance their self-efficacy (Westman *et al*., 2004). Resources are critical to combat loss, recover lost resources and protect against future loss of resources. A suite of resources such as formalised HRM practices may play a pivotal role in preventing loss or threat of loss, as well as recovering from resource loss (Bamber *et al*., 2017). Hence, workers are likely to use their accessible resources (resource gain), leading to resource caravans and gain spirals (Hobfoll *et al*., 2018). However, a lack of access to a pool of resources may lead to a loss spiral resulting in declined commitment due to the depletion of resources (Hobfoll *et al*., 2018).

Resource caravans aim to provide workers with shared resources to promote the accumulation of a supportive environment and the integration of resources (Hobfoll, 2011) to protect against resource depletion and mental health challenges. For example, if nurse managers, nurses and PCAs were provided with management support and HRM practices, they would be able to integrate resource caravans to mitigate the negative effects of workplace violence. Consequently, this may influence positive organisational outcomes (e.g. employee engagement and job satisfaction). A foundation for gain spirals can enhance the effects of resources through continual and consistent resource inputs (Lapointe *et al*., 2019), which contributes to more active acquisition of resources. Lapointe *et al*. (2019) argue that resource deficiencies have an impact on the health and well-being of workers. On the other hand, gain spirals contribute to less vulnerability of workers and allow them to invest more resources to cope with job demands (e.g. negative effects of workplace violence). Therefore, the use of resource gain, resource caravans and gain spirals can help minimise the effects of negative work events that impact the mental health of healthcare workers (Hobfoll *et al*., 2018).

### 3.1 Bourdieu’s Theory of Practice (ToP)

We drew on Bourdieu’s ToP (1977) to examine the symbolic violence of management in a work environment where workplace violence is accepted by nurse managers and staff as “normal” (Cavanagh *et al.*, 2021). ToP adds to a combined framework with COR theory to understand social actions at individual and organisational levels (Bourdieu, 1977). Central to Bourdieu’s theory is a set of circumstances comprising field, capital, habitus and doxa, which enables systematic examination of social order within a given “field” that underpins and fosters symbolic violence.

Aged care facilities are the “field” and a social space within a workplace (Bourdieu, 1985), and in this study, they comprise aged care facilities. An individual’s position on the “field” depends on their capital, which determines an individual’s position and advantage (Harrington *et al.*, 2015). Social capital comprises the participants, nurse managers, nurses and PCAs, and the relationships they have at work that influence their position in the field. This may reveal itself in the cultural capital of workers, for example, the institutional (education) and symbolic (prestige) capital held compared to other members of staff (Bourdieu, 1986; Ocasio *et al.*, 2020). For example, PCAs may be lower on the aged care facility hierarchy than nurses due to their lower educational standards and lower standing within the field (Cavanagh *et al*., 2021).

Habitus is defined as “durable, transposable dispositions” of actors within a field (Bourdieu, 1977, p. 53). Habitus refers to the inclination and propensity to behave in a certain way (Bourdieu, 1977). Aligning oneself to the habitus within a field will usually bring an individual social and cultural capital (e.g. respect and status). Through shared habitus, social practices become immediately understandable and predictable to similarly situated individuals, especially within a field. An important outcome of shared habitus is that individuals become less critical of their own and each other’s behaviour as they are subsumed in the logic of the field (Bourdieu, 1977). This may be manifested in the acceptance of workplace violence in aged care facilities for workers to “get on” in the workplace.

Doxa is explained as the underlying and unquestioned beliefs and assumptions commonly held by actors in a field (Bourdieu, 1977). The doxa of a field is socially constructed and accepted as the norm by actors and provides the organisation’s ultimate basis of power. The power of management in effect creates an acceptance by subordinates of doxa as “normal”, which in some instances should be questioned (e.g. workplace violence as part of the job role). Bourdieu (1985) suggests that dominators (e.g. managers in the aged care facilities) may defend their positions within the hierarchy, asserting the worth of whatever capital typifies their habitus or improving their position within the social order. In aged care facilities, this may demonstrate how managers increase profits and reduce workplace injury insurance claims.

Symbolic violence is characterised and played out by the senior management of organisations when they reinforce constraints on workers and deny them their voice, promotion opportunities and/or any autonomy. Management does this by dominating workers through subtle ways of imposing their values, beliefs and norms on staff (Bourdieu, 1977). Workers will often accept the views of management as legitimate and even though they are aware that this is a form of discrimination and domination, workers will not question management for fear of retribution (Bourdieu, 1977). The unequal power relations silences workers, and they accept the authority of management. Symbolic violence impacts on workers sense of belonging in a workplace and fosters a culture of inequality. Workers then tend to be accepting of their own marginalisation in the workplace.

## 4. Methodology

Our study took a qualitative case study approach to carry out in-depth interviews with 60 participants made up of 10 nurse managers and 50 nurses and PCAs working in aged care in Victoria, Australia. The Australian Nursing and Midwifery Federation (ANMF) approved the case study approach to better understand complex work-related issues around the daily work activities of managers, nurses and personal care assistants working in aged care facilities. The scope of the study involved public, private and not-for-profit aged care facilities across metropolitan, regional and rural areas of the state. A case study approach provided the researchers with an effective method to unpack participants’ in-depth and profound insights into the phenomenon of work that a quantitative method cannot achieve (Silverman, 2020). We aimed to achieve an interpretative and naturalistic approach to the research (Lofland *et al*., 2006) to understand the workplace experiences of nurse managers, nurses and PCAs and their views of working in aged care. Participation was voluntary and identifying participant information was anonymised from the point of transcription (Creswell, 2009). The research was designed as a case study to investigate the research problem of workplace violence against nurses and PCAs in the aged care sector. A case study approach of this nature can include participants from various organisations in the one sector (e.g. aged care) when there is a common complex issue (e.g. workplace violence) in a real-life context (Yin, 2014; Creswell, 2009). Data were collected through interviews (Yin, 2014) and analysed with NVivo (Weber, 1990) to identify key themes. This case study was informed by COR (Hobfoll, 1989) as the theoretical framework to examine incidents of resident-on-nurse/PCA violence.

### 4.1 Study setting and recruitment

The study was carried out with participants comprised of 10 nurse managers (NM), 50 nurses and PCAs. The nurses were made up of 13 registered nurses (RN), 14 enrolled endorsed nurses (EENs) and 10 enrolled nurses (EN), and throughout the paper, they were collectively referred to as “nurses” and there were 13 PCAs who were all members of the ANMF and referred to as PCAs – see Table 1. The study was promoted via the occupational health and safety (OHS) coordinator of the ANMF, who liaised with prospective participants and disseminated a recruitment message via a fortnightly email newsletter to ANMF members. Nurse managers will be reported as Yasna (NM, Public); a nurse who works in a private aged care facility will be referred to as Benedicta (RN, Private); a PCA who works in a not-for-profit aged care facility will be referred to as Ana (PCA, NFP). Our study did not set out to compare the perceptions of participants across public, private and not-for-profit facilities. The main focus was to capture patterns of meanings from nurse managers, nurses and PCAs personal perspectives (Creswell, 2009). Prospective participants received a participant information statement outlining the aims and benefits of the research project and invited interested participants to sign a consent form and return it to the researchers via email (Glesne, 2016). Participants were only contacted once signed consent forms were received by the researchers.

**Table 1:** Participants

Aged care workers	Pseudonyms and role	Total	Type of aged care facility
4 Nurses and 3 PCAs	Daniela (RN), Alicia (EN), Emma (EEN), Monica (RN)	7	Public aged care facilities
	Lucia (PCA), Adriana (PCA), Felipa (PCA)		
16 Nurses and 4 PCAs	Erika (EEN), Renata (EN), Fabiola (EEN), Seferina (RN), Leslie (EEN), Julie (RN), Benedicta (RN), Mayra (EN), Sofia (EN), Eva (EEN), Emily (RN), Mia (EN), Rachel (EEN), Therese (RN), Susana (EEN), Alessia (EN)	20	Private aged care facilities
	Luana (PCA), Paula (PCA), Maria (PCA), Alejandra (PCA)		
18 Nurses and 5 PCAs	Ava (EEN), Isla (EN), Carlota (RN), Olivia (EEN), Amelia (EN), Milly (EN), Lidia (RN), Ines (EEN), Micaela (EN), Annie (EN) Marie (PCA, Isabel (RN), Gloria (EEN), Leonor (EEN), Quinnie (RN), Juliana (RN), Josefina (EEN), Fernanda (RN)	23	Not-for-profit aged care facilities
	Ana (PCA), Julia (PCA), Revoreda (PCA), Marta (PCA), Pamela (PCA)		
		50	
*Nurse Managers*		*Total*	*Type of aged care facility*
3 Nurse Managers	Yasna (Nurse Manager), Jenny (Nurse Manager), Nancy (Nurse Manager)	3	Public aged care facilities
4 Nurse Managers	Nina (Nurse Manager), Jana (Nurse Manager), Rose (Nurse Manager), Mary (Nurse Manager)	4	Private aged care facilities
3 Nurse Managers	Rita (Nurse Manager), Victoria (Nurse Manager), Irina (Manager)	3	Not-for-profit aged care facilities
		10	

### 4.2 Data collection

Data were collected from the 10 nurse managers and 50 nurses and PCAs who work in aged care facilities in the public, private and not-for-profit sectors. Prior to collecting data, the researchers met and reflected on their own experiences and any potential biases. When collecting the data, we were mindful that interview questions require the researchers’ creativity, flexibility and insights into a phenomenon to encourage participants to share their experiences (Seidman, 2013). Hence, it was decided that a degree of flexibility would be exercised during the interviews to give the researchers the opportunity to expand on any issues arising from participants’ narratives.

Interviewing is a powerful way to gain insights into “the lived experience of participants and the meaning they make of that experience” (Seidman, 2013, p. 9). Each interview was conducted in person or via telephone, and each interview was for 50–60 minutes. The interviews conducted in person were attended by two interviewers. In addition to the main research question, here are some of ancillary questions: What are the perspectives of nurse managers, nurses and PCAs concerning the impact of workplace violence on their mental health and well-being? What are the implications of workplace violence for nurse managers and HRM departments in aged care facilities? If a participant wanted to expand on a question, we welcomed their perspectives. We examined the main research question, and these questions were based on 60 interviews with nurse managers and nurses and PCAs working in aged care facilities across Victoria, Australia.

Interviews were audio-recorded and transcribed verbatim using the services of an external company. Researchers maintained field notes to compare transcriptions and ensure accuracy. To prepare for the data analysis phase and ensure reliability and validity of the data, the researchers worked in pairs and regularly discussed interpretations of the data collected and notes taken during the interviews (Yin, 2014; Creswell, 2009; Lincoln and Guba, 2000).

### 4.3 Data analysis

Computer-assisted qualitative data analysis software NVivo was employed to conduct a thematic content analysis and identify themes in the data (Weber, 1990) to search for patterns in the responses from nurse managers, nurses and PCAs. The software assists in categorising data to respond to the research question and sub-questions. According to Yin (2014), pattern matching aims to identify patterns of variables within a singular or multiple case study. After themes are identified, they support the analysis and interpretation of data (Weber, 1990) provided by nurse managers, nurses and PCAs. The final coding framework and analyses of interview data are read by two coders to ensure reliability (Weber, 1990). In NVivo we entered code root levels which expanded into sub-trees of five words. For example, when we entered the root level “workplace violence” this branched into five subtress of “physical violence”, “verbal violence”, “harassment”, “bullying” and “aggression”. Checking transcripts for data saturation contributed to rigour in the data collection, data coding and data analysis (Creswell, 2009). We reached data saturation when we heard the same stories repeated, and there were no new themes arising from the interviews (Yin, 2014). The researchers discussed each of the themes to determine how each researcher interpreted and evaluated the same data on each theme. When there were variations of interpretation, the researchers continued to discuss the themes until they reached similar conclusions (Seidman, 2013).

### 4.4 Ethical and research considerations

Ethical considerations are critical to any research project (Yin, 2016). The researchers obtained ethical clearance from the University Human Research Ethics Committee under Project No. 21961 dated 17th May 2019.

The researchers are not nurses or PCAs and have never been employed in aged care facilities. The researchers have a strong track record in publishing in HRM and healthcare/nursing management and have a collective of 264 publications in national and international refereed journals, including multiple articles in the Journal of Advanced Nursing. One of the researchers is a psychologist, one is a professor who has over 25 years of research experience in human resource management and healthcare management, and the third researcher has extensive industry (legal) experience and 20 years of research in human resource management and healthcare management. We agreed that it was important to approach the research without any assumptions and allow the participants and the data to tell their stories.

## 5. Findings and analysis

In this section, we present the findings and analysis through identified themes. Five themes emerged from the data related to nurse managers, nurses and PCAs: first, incidents of workplace violence; second, managers perspectives on the management of workplace violence; third, the demands on nurse managers, nurses and PCAs; fourth, perceptions of nurse managers, nurses and PCAs of a dearth of resources; and fifth, nurse managers and nurses and PCAs and the impact on their mental health. Our study found that the participants interviewed had been impacted by workplace violence and symbolic violence (Bourdieu, 1977). There were similarities in participants' accounts of the type of workplace violence, how violence impacted their mental health and well-being, and their perceptions of how incidents were managed. An interesting finding was that every one of the participants talked about how their facilities are under-resourced which is one way that senior management imposes symbolic violence (Bourdieu, 1977). Nurse managers, nurses and PCAs all commented on how their facilities are understaffed and how it is challenging to provide consistent high-quality care for residents, which may exacerbate incidents of violence.

### 5.1 Incidents of workplace violence experienced by nurse managers, nurses and PCAs

When we asked nurse managers about daily work, they were unsurprised by questions about workplace incidents. Yasna (NM, Public) said *“violence happens daily towards staff … I’ve experienced bad language but nothing physical … it’s mostly secondary stress for me”*. *“I get involved in serious incidents ….for example, last week a male resident tried to push a nurse out the window … it’s part of the job”* (Nina, NM, Private). Nurse managers made it clear that workplace violence happens “daily” in aged care facilities, and from their perspectives, violence is *“part of the job”.* Nurse managers’ experiences were more around *“bad language”* from residents. Nurse managers indicated that their priority was to *“run the facility”*, *“process residents”* and carry out *“administration”*. Bourdieu (1977) views this as a domination technique, imposing symbolic violence and weakening workers’ position in the workplace.

We asked less direct questions of nurses and PCAs about their daily work, yet the participants told multiple stories about incidents of violence that occur every day in aged care workplaces. Erika (EEN, Private) explained how residents use derogatory language whilst physically hurting staff *“ …..the words they* [residents] *use puts you down …. They’re also pinching you and grabbing at you”.* Similarly, Ana (PCA, NFP) explained frequent experiences of violence *“We had a lady …. she would … run up behind us and grab our hair ….I got no* [management] *help”.* The nurses and PCAs were consistent in their accounts of violence that impacts their personal resources and drives their reduced performance, stress and burnout (Hobfoll, 1989). Nurses and PCAs experience serious incidents of physical violence daily. They talked about being “*physically assaulted* [by residents]” and *“traumatised”* by serious incidents such as when a resident “broke the jaw” of a PCA. There was disappointment in the voices of nurses and PCAs as they expressed how management will often *“ignore staff”* when incidents of workplace violence happen (Pariona-Cabrera *et al.*, 2020). Nurses and PCAs appeared to rely on their own resources to deal with adverse work events (Hobfoll, 1989) and indicated this was due to their perceptions of a lack of management support and poorly enacted anti-violence HRM practices.

### 5.2 Nurse managers perspectives on the management of workplace violence

The nurse managers told interviewers they are supportive of workers in aged care facilities but within the scope of their roles which Jana (NM, Private) explained *“I only get involved in the serious cases* [of workplace violence] ….*my job is excessively administrative ….there’s lots of government reporting and processing residents is our business ….I know there’s lots of pinching, punching and name calling ….”.* Rose (NM, Private) mentioned *“in every facility staff are expected to report incidents of violence ….staff are too busy ….much of it goes unreported”*. Managers were willing to share some of the stories, but their focus, according to Yasna (NM, Public), was on how it was to keep staff working after an incident of violence. It was evident that nurse managers' mental health was impacted by “*stress”*, *“burnout”* and *“anxiety”.* After sharing incidents of violence against staff, managers avoided discussing details on the mental health of these workers. Nurse managers explained that their roles are complex and that their priorities are focused on sustaining facility operations and the continuity of care for residents. Bourdieu (1977) would argue that habitus highlights how senior management maintains control over nurse managers to avoid supporting workers. While managers acknowledged workplace violence was a common occurrence in aged care facilities, they indicated that many incidents are *“under reported”* because workers *“don’t have time”* to report every incident of workplace violence. Managers did not appear to prioritise the impact of workplace violence on staff, and Hobfoll *et al*. (2018) expect this could lead to a loss of commitment of staff towards their work, and in this case, important care work. Watson and Hatcher (2021) argue tensions then occur between management and staff when staff perceive that their concerns are diminished or ignored. All managers of this study confirmed their concern was to ensure staff *“keep working”* (following an incident of workplace violence), which indicated they may need to consider better ways of managing negative work events and the impact of workplace violence on the mental health and well-being of workers (Hobfoll *et al*., 2018).

### 5.3 Demands on nurse managers, nurses and PCAs

Managers talked broadly about their responsibilities and the many demands on their roles. COR theory (Hobfoll, 1989) emphasises how demands can deplete resources. Rita (NM, NFP) told the researchers, *“I have to keep operations of the facility running smoothly … lots of administrative and care [resident] responsibilities … no time to think”*. Jenny (NM, Public) explained *“I manage the facility and the staff ….but I also have to look after residents … at times it feels I’m overloaded”*. We found evidence that job demands (Hobfoll, 1989) and negative work events happened every day for the nurse managers, nurses and PCAs of this study (Zhang *et al*., 2021). The demands on managers include maintaining the operations of each facility in line with government standards and reporting requirements and ensuring the continuous care of residents. Nurse managers are under the control of senior managers who have high demands (Hobfoll, 1989), which means they transfer those demands and impose symbolic violence on workers (Bourdieu, 1977). We identified a tension when it came to management responding to workplace violence events (Watson and Hatcher, 2021) in a supportive and caring way because managers’ focus was on maintaining effective operations of aged care facilities to satisfy the demands of senior management (Hobfoll, 1989).

Participants elucidated how the demands on PCAs are excessively high, which clarifies Hobfoll’s (1989) theory that lower-level workers are at a higher risk. PCAs constantly told us how senior management and nurse managers do *“not value”* them and, in fact, *“disrespect”* them. Marta (PCA, NFP) voiced her disappointment about management *“ … about 73% of aged care staff are PCAs but management treats us as shit. I would like every one of us, on one day, to stand up and say we won’t come to work”*. The participants also expressed their disappointment that administrative staff recognise PCA work as *“dangerous”*, *“stressful”* and *“unsustainable”* but *“management doesn’t care”.* Lucia (PCA, Public) described *“the biggest challenge at work …..is getting management to listen … and we need more staff”.* Work pressures and a lack of staff were reported by many participants as having a substantial demand on them as workers (Hobfoll, 1989). Heavy workloads and inadequate staffing experienced by nurse managers, nurses and PCAs were consistently found to contribute to mental health challenges. These circumstances are typical of senior management imposing symbolic violence on lower-level staff to control them (Bourdieu, 1977). For aged care nurses and PCAs, job demands involved dealing with daily incidents of workplace violence, which were exacerbated due to the perception of managers and HRM departments of *“not providing support”.* There was evidence that aged care facilities often have outsourced HRM services and do not have a visible presence in the day-to-day operations of the aged care facilities (Cavanagh *et al.*, 2021).

### 5.4 Perceptions of nurse managers, nurses and PCAs on the lack of resources

Nurse managers of this study claimed they do not have the resources to support staff and Rita (NM, NFP) highlighted their collective views that staff ratios are not adequate for the number of residents, *“if we had more staff, we could spend more time on their* [resident]*care”.* In the interviews with nurse managers, we heard that HRM is external and when they are involved in staff-related issues in aged care facilities, *“it turns into a monstrous mess … because they* [senior management] *don’t provide resources or support”* (Mary, NM, Private). Senior management minimises resources (Hobfoll, 1989) as a way to exercise symbolic violence and control workers (Bourdieu, 1977).

Every one of the participants narrated multiple stories about how they try to retain resources (Hobfoll, 1989). However, they are expected to perform their work with *“limited resources”*, *“unreasonable demands”* and at the same time are subjected to adverse physical and verbal incidents from residents. The ratio of nurses and PCAs to residents was raised as an issue repeatedly throughout the interviews, because *“there’s not enough staff”.* As well, nurse managers perceived that HR departments are *“not visible”* and did not provide the support they needed, especially in terms of anti-violence HRM practices. There was often a lack of perceived senior management support and time to get the job done, which contributed to workplace challenges for managers.

When we asked participants about incidents of violence Daniela (RN, Public) explained *“ ….it’s belittling and hard especially when you face negative situations at work”* and managers *“ ….dismiss you … makes me feel worse when managers do nothing”.* Luana (PCA, Private) explained *“ ….they’re* [managers] *more worried about doing paperwork and ticking boxes …. managers don’t give* [staff] *any backup or support at all”.* Our study found little management support for workers. COR theory (Hobfoll, 1989) contends that a lack of management support may contribute to a loss of social resources, which may exacerbate job stress. When nurses and PCAs do not receive support, they are inhibited by not feeling in control, and many of them talked about how they can be easily replaced – *“we’re dispensable”, “they* [management] *can replace us”* and *“management will find someone to replace me by tomorrow”.*

All of the participants told us they were so stressed and exhausted and did not have the energy to be empathetic to residents. Participants unanimously expressed negative feelings about the adverse impact on their work relationships with coworkers and residents (Cavanagh *et al.*, 2021). Their views were consistent about a lack of management support when staff face adverse work events. Participants also claimed there was a deficiency of staff, which impacted their ability to perform in their role (Hobfoll, 1989). Many participants told us they did not “*have time”* to report every incident of violence.

### 5.5 Nurse managers, nurses and PCAs and the impact of workplace violence on their mental health

The researchers asked questions about how and why workplace incidents impact on managers. Mary (NM, Private) shared *“ … everyone* [staff, residents, families] *has complaints, requests, concerns ….I’m one person …..it’s emotionally overwhelming”*. *“We have to find solutions to all kinds of workplace issues … sometimes I just can’t cope”* (Nina, NM, Private). Nancy (NM, Public) highlighted *“I don’t receive support from the hierarchy … I’m a human being and it’s hard to hear staff stuff* [workplace violence] … *stresses me a lot … I struggle to control my emotions”.* While the managers in our study indicated they do not experience workplace violence to the same degree as nurses and PCAs, they indicated they are impacted by daily reports of violence against their staff and balancing care for residents. Managers found it *“emotionally overwhelming”* and they often *“just can’t cope”* which is an indication of resource loss (Hobfoll, 1989). Ruiz-Fernández *et al*. (2020) argue healthcare professionals experience high levels of job stress when they must deal with traumatic and complex work situations with residents.

We examined the perspectives of nurse managers, nurses and PCAs on the effects of workplace violence on their mental health and well-being. Fabiola (EEN, Private) expressed *“ …..punching, biting, spitting, swearing* [by residents] ….*gives me an overwhelming sense of anxiety …..very threatening”.* Alicia (EN, Public) told us *“It* [violence] *impacts on our work and care with residents because we’re under stress and we’re exhausted”.* Seferina (RN, Private) explained *“I have nightmares and flashbacks every day and I’m unable to cope”*. Workers described how they felt, with words such as *“panicky”, “scared”, “frightened”* and *“anxious”* when they told stories about workplace violence. The participants expressed unanimous feelings of stress and fatigue (resource loss) after experiencing workplace violence and how this impacted negatively on their mental health (Pariona-Cabrera *et al*., 2020). Many participants reported how much time it took them to recover from workplace incidents because of poor management support (symbolic violence) and how incidents result in mental health issues. They constantly experience negative feelings that leave them wanting to resign from work. Our findings highlight incidents of workplace violence that impact the mental health of nurses and PCAs and leave them with feelings of stress and fatigue, which negatively impacts their mental health.

Table 2 provides a summary of the themes and additional narratives from participants:

**Table 2:** Themes

Themes	Participant narratives aligned to the themes
*Incidents of workplace violence experienced by managers, aged care workers*	“*Violence happens towards the staff ….not so much managers … but, when I’m called to an incident my main concern is how to settle the resident … ”* (Victoria, NM, NFP)
	*“ … I was verbally abused last night … he* [resident*] called me a bastard … and one of my friend’s hands was destroyed … because of repetitive twisting of the hand and wrist by a resident … ”* (Monica, RN, Public).
	*“I’m on Workcover* *at the moment, due to violence … I’ve been physically assaulted on many occasions”* (Leslie, EEN, Private)
	*“ … one particular incident … I was quite traumatised …. a resident actually broke my jaw”.* (Maria, PCA, Private)
*Nurse managers perspectives on the management of workplace violence*	*“I don’t have time to hear about every little incident … I wouldn’t get any work done … management and administrative responsibilities are challenging and time consuming … ” (*Nancy, NM, Public)
	*“Violence happens every shift, every single day … it’s like we expect it … they [staff] expect it … I guess then it’s not always dealt with because staff don’t have time to report every incident”* (Irina, NM, NFP)
*The demands on nurse managers and aged care workers*	*“Because of the ratio of staff to residents the shifts can be hard, and I used to work … with up to 32 residents to one nurse. You feel burnt out every day”* (Renata, EN, Private)
	*“Workload has a big impact because we can’t handle it ….managers have to be taught how to care for their staff”* (Isabel, RN, NFP)
	*“I should be working 7* *h per day ….but, every single day I work another 2 to 4* *h … I’m stressed, exhausted, and fatigued from work”* (Micaela, EN, NFP)
	*“ … another nurse said she would rather shoot herself in the head than be a PCA”* (Ava, EEN, NFP)
	*“We are short staffed, and they* [management] *don’t replace staff who leave”* (Leonor, EEN, NFP)
	*“ ….there’s not enough staff to help out …. rush, rush, rush ….you never get to slow down or stop … this is stressful”* (Pamela, PCA, NFP)
	*“ … we can lose a resident on a Friday and have a new resident in there on Monday … you just have no time to process anything”* (Susana, EEN, Private)
Perceptions of nurse managers, aged care workers of a dearth of resources	*“We don’t always tell them* [management about workplace violence] *because they simply don’t want to know”* (Emily, RN, Private)
	*“I’m feeling really overwhelmed, stressed and very irritable … this impacts on staff self-esteem and …..with other people* [colleagues, residents]*”* (Emma, EEN, Public)
	*“After experiencing these negative events, I feel all the negative emotions … stress, anxiety, and exhaustion”* (Julia, PCA, NFP)
	*“When I am physically attacked* [by a resident] *… I don’t want to go back to work again”* (Rachel, EEN, Private)
Nurse managers, aged care workers and the impact of workplace violence on their mental health	*“ … our staff experience violence every day and this cause me stress because I just can’t fit it”* (Irina, NM, NFP)
	*“Working with people with dementia is difficult ….I recently took three-days sick leave … I can’t work anymore”* (Lidia, RN, NFP)
	*“ ….at the end of every shift we’re all stressed, burnt out and exhausted ….never stops …. I’m tired of being nice to residents … ”* (Paula, RN, Private)
	*“I feel extremely vulnerable, and it gets worse with every shift. Any slight noise at work is likely to startle me”* (Josefina, EEN, NFP)
	*“I’m stressed and then emotionally drained …..you wake up in the morning thinking, why should I bother going to work?”* (Gloria, EEN, NFP)
	*“It just kills me every day … I feel stressed and exhausted … I have to leave this profession”* (Alejandra, PCA, Private)

## 6. Discussion

Aged care environments are characterised by high emotional demands, unpredictable resident behaviours and constant workload pressures, all of which threaten staff resources. We examined the ways in which incidents of workplace violence against nurse managers, nurses and PCAs impacted their mental health and well-being. Underpinned by COR theory (Hobfoll, 1989), we respond to the unique findings of nurse managers, nurses and PCAs on their experiences of workplace violence and the impact on their mental health and well-being. Notably, workplace violence affected every one of the 60 participants, but there were clear differences between how violence impacted nurse managers compared to nurses and PCAs. Nurse managers reported feeling overwhelmed by their daily responsibilities and having to deal with complaints (about repeated incidents of violence) from nurses and PCAs while they were maintaining the full operation of aged care facilities. In effect, nurse managers are controlled by senior management and challenged when nurses and PCAs report incidents of violence.

We found that senior management, either intentionally or unintentionally, imposes symbolic violence on workers and normalises violence in aged care facilities through a number of different but consistent mechanisms. The interconnectedness of the following mechanisms contributes to the normalisation of workplace violence in aged care facilities. First, managers tend to prioritise budget constraints and the delivery of aged care services to specific targets (e.g. staffing ratios) and ignore staff safety (Bourdieu, 1977), which is an indicator that workplace violence is not important and even acceptable. Second, when managers make light of incidents or ignore episodes of violence as being an accepted “part of the job” they effectively redefine “harm” as an acceptable behaviour (Bourdieu, 1977). Third, the fact that managers do not encourage staff to complete incident reports is a covert message that staff might be to blame for workplace violence (Bourdieu, 1977). Fourth, following incidents of violence, managers do not offer psychological support for staff, again signalling that workplace violence is not important enough to warrant hierarchical action. Fifth, in aged care facilities there is a culture of silence amongst staff and a deliberate reluctance to have a voice, often due to a fear blame. Collectively, through symbolic violence, these mechanisms create a workplace environment where violence is tolerated and becomes normalised in everyday practice. The hierarchy exercises symbolic violence (Bourdieu, 1977) through the abovementioned mechanisms to ensure there is little management support systems for workers, limited resources, and by imposing low staff-to-patient ratios (Pariona-Cabrera *et al*., 2020).

Nurses and PCAs arrive at work every day knowing there will be some level of verbal and/or physical violence yet, at the same time, not knowing exactly how or when violence will occur. The tensions between knowing and not knowing mean nurses and PCAs have to be vigilant in how they approach their daily work, which also creates an undercurrent of fear and anxiety that can lead to burnout (Bartram *et al*., 2023). In these circumstances, workers report they have a sense of helplessness that consequently impacts their mental health and well-being. Participants’ stories highlighted incidents when they endured physical violence, such as *“repetitive twisting of the hand”*, *“pinching”* and *“punching”* and verbal violence when residents call staff *“a bastard”* and these incidents are considered the norm in aged care facilities. When nurses and PCAs experience incidents of violence, repeated incidents, their resources decline (Hobfoll, 1989), and there is a negative impact on their mental health of workers (Pariona-Cabrera *et al*., 2020). Often these workers do not want *“to go back to work”* particularly when they know they will not be supported by management (Bourdieu, 1977). All of the participants expressed their concern about withdrawing compassion for the residents they care for, *“I can’t keep caring”, “it’s difficult to empathise”* and *“I’ve lost my motivation”* (for residents). This is in line with COR theory, suggesting that workers will withdraw their labour to minimise further loss of resources (Hobfoll, 1989). According to Bourdieu (1977), doxa explains the unquestioned beliefs of nurses and PCAs that violence is an accepted norm under management that has the power to control them. There was no management support for nurses and PCAs against workplace violence or implementation of anti-violence HRM practices (Shao *et al*., 2023). We found some evidence in the data that this may influence nurse managers, nurses, PCAs’ intention to leave aged care facilities.

### 6.1 Contributions to theory development

Our paper makes an important contribution to management and HR management literature in the context of aged care facilities and the mental health consequences of workplace violence on the mental health and well-being of nurse managers, nurses and PCAs. Our contribution highlights how the normalisation of violence in the aged care sector is shaped by senior management through symbolic violence and a reluctance of nurses and PCAs to challenge management decisions and authoritarian control. We approached the mental health challenges by examining the perspectives of nurse managers, nurses and PCAs. To address these challenges, the current focus on resource loss has to be reversed to facilitate the reversal of recourse loss into resource gain. It will be through resource gain that stress levels can be better managed to promote the psychological well-being of managers and workers. Workers are more likely to be more motivated to build and retain resource gains when there are mechanisms in place that focus on resource gains that support their mental health.

Our study contributes to the theoretical relevance of Hobfoll’s COR theory by demonstrating how it can be relevant to contemporary workers and organisations within a specific context or sector. The value of COR theory (Hobfoll, 1989) is important to our study because it provided a lens to better understand the process through which workplace violence erodes the personal resources of workers, such as nurses and PCAs. However, COR theory has been criticised because of its focus on generic resource loss cycles and the limitations around the motivation and resilience of individuals to manage stress by gaining and protecting resources (Lazarus, 2001). We challenge the theory to highlight its transformative potential by taking resource loss cycles and converting them into resource gain cycles. Transformative change can better support the increasing mental health challenges of many workers due to incidents of aggression and violence at the workplace (e.g. nurses and PCAs of this study, as well as doctors, paramedics, police, hospitality workers, teachers). To reverse negative resource loss cycles, the theory would require a definition of “resources” in a specific context and the degree to which workers experience aggression and/or violence at work, and any negative mental health consequences (Halbesleben *et al.*, 2014). While COR theory highlights the lack of management and HRM support, which compounds the negative effects of job demands such as workplace violence on staff, it should be supported with leadership engagement. This requires deep management-level understandings and support for workers and how they are exposed to and respond to resource risks. The concept of stress must go beyond responding with resilience (as per COR theory) and include mental health repair and recovery (Sonnentag and Meier, 2024). To support mental health repair and recovery, incorporate digital resources and interventions such as apps and mental health programmes that may sustain the mental health and well-being of workers (Roquet *et al.*, 2023).

### 6.2 Implications for policy and practice

There are implications for nurse managers to develop four-tiered policies and procedures to prevent incidents of violence against nurses and PCAs, provide support for the ongoing impact of incidents of violence and mitigate factors that drive potential turnover. First, the nurses and PCAs claimed they did not receive orientation prior to commencing work. They were basically *“thrown into the deep end and had to swim or drown”.* Second, nurse managers need to be more aware that nurses and PCAs, who are exposed to occupational stressors (e.g. resident violence), are at a higher risk of developing mental health issues (Ruiz-Fernández *et al*., 2020). When nurses and PCAs report incidents of violence, management should have a well-being policy and provide comprehensive reporting systems with well-documented procedures in an attempt to alleviate incidents of violence. Third, there needs to be policy and procedures that ensure ongoing support that acknowledge the potential impact of violence against the mental health and well-being of all employees. Such support systems would include ways to decrease incidents of violence (e.g. ensure two staff members always work together with residents) and provide strategies to boost resilience against stress and burnout. A well-being policy would set out the elements to promote mental health and well-being through awareness, coping mechanisms and organisational care for nurses and PCAs. Training and development plans could incorporate a training needs analysis to identify the needs of nurses and PCAs around mental health, training sessions with set objectives and activities to better manage mental health issues and regular evaluation processes. Fourth, nurse managers need to ensure policy and procedures to better manage the impact of long-term violence against nurses and PCAs that drives their decisions to leave employment. Nurse managers need to implement retention strategies that guarantee fair staff ratios to residents, provide safe work environments and include mental health and well-being programmes.

We encourage nurse managers to be more aware and respond to the Royal Commission into Aged Care Quality and Safety (2021) report to better support the aged care workforce and commit to “a highly skilled, well rewarded and valued aged care workforce … ” (p. 124). Importantly, HRM professionals need to have strong working relationships with managers in their aged care facilities (e.g. notable facility presence). When senior management support and HRM systems are either not in place or effectively communicated and understood by managers, they may be overwhelmed with the responsibilities of their roles, such as the management and mitigation of workplace violence (Pariona Cabrera *et al*., 2023).

Nurses and PCAs’ perceptions of the support they receive from the organisation, nurse managers, HR departments and other staff members are important in the determination of resources available to mitigate the negative effects of job demands, such as workplace violence and conserve resources. The perceptions of aged care staff will be influenced by the extent to which they perceive job demands (e.g. high levels of physical violence may be perceived differently relative to verbal violence). This has important implications for nurse managers and HR departments in that it informs them to be conscious about communications and staff understandings and interpretations of the job resources that are available to them (Bowen and Ostroff, 2004). HRM departments and managers in aged care facilities also need to ensure that health and safety compliance is followed in accordance with relevant legislation and employment agreements. It is the responsibility of managers and workers to ensure that incidents of violence are reported, and measures are taken to avoid further workplace violence incidents.

### 6.3 Limitations and recommendations for future research

Our study is not without limitations. Non-ANMF members were not included as a comparison group in this study. Therefore, it was not possible to determine if the experiences raised by nurse managers, nurses and PCAs in aged care facilities were unique to this sector. While we interviewed nurse managers, nurses and PCAs from public, private and not-for-profit aged care facilities, we did not set out to compare the three sectors. We did not interview HR managers and note this as a limitation and recommend that a future study include the perspectives of these managers. We note that participants may well have other reasons for mental health challenges, such as personal coping, team issues, family or other external matters that may cause them stress. Our study did not examine the annual wage review decision announced in June 2023, which meant an increase in wages by 5.75% (Department of Health and Aged care, 2023). We acknowledge that this could have represented an interesting avenue for research to ascertain the effects of increased wages on the attraction and retention of nurse managers, nurses and PCAs. Moreover, our study did not examine the role of power or its use within aged care facilities or the effects of healthcare systems and the organisation of care for residents. This may be an important research avenue in the future.

We recommend a mixed methods study including stakeholders such as nurse managers, nurses, PCAs, HR managers and medical professionals situated in aged-care facilities (e.g. doctors, physiotherapists, nutritionists). This proposed study would examine and compare causes, effects and solutions to workplace violence through the perspectives of the different stakeholders in organisational settings. This type of research could further the understanding of the lived experience of key stakeholders and their day-to-day interactions in aged care facilities. We recommend that future research examine the power dynamics between the hierarchy and managers in aged care facilities. Researchers may also consider different healthcare systems, aged care ownership structures and the impact they have on workers, their mental health and care for residents. Furthermore, given the annual wage review decision announced in June 2023, meant an increase in wages by 5.75% (Department of Health and Aged care, 2023) we recommend further research to ascertain the effects of increased wages on the attraction and retention of nurses and PCAs and the effects on their mental health and well-being. Our study did not examine the international context, and we recommend future research on workplace violence and its effects on nurse managers, nurses and PCAs in different national contexts (e.g. developed and developing countries and a variety of cultural and religious contexts) and across public, private and not-for-profit aged care facilities. We also suggest examining workplace violence in other contexts such as hospitals and other healthcare services (e.g. psychology, physical therapy and radiography).

## 7. Conclusion

Our study has argued that the nurse managers of the aged care facilities in this study should be more engaged with HR departments and work towards eliminating adverse well-being cycles through a broad range of HRM practices and activities (e.g. post-incident debriefings and counselling for staff) to ensure the retention of nurses and PCAs in the workplace. We strongly encourage nurse managers to invest in anti-violence HRM practices (e.g. management interventions pre-and-post incidents of violence, anti-violence training and security measures) to better support the mental health and well-being of nurses and PCAs. An important part of combatting workplace violence is compliance with health and safety legislation and preventative HRM policies around negative workplace behaviours. Given the continuing challenges of workplace violence in aged care facilities and the impact of mental health and well-being on managers and staff, we encourage aged care nurse managers and HR departments to focus on enhancing the employment experience of staff and the care of residents. Due to an escalating demand for aged care services and the projected decline in aged care nurse numbers in the future, much more needs to be done to support the mental health and safety of all workers employed within aged care facilities.
